# Supplementary material for: Modeling glioblastoma heterogeneity as a dynamic network of cell states
Source: Mol Syst Biol. 2021 Sep 16;17(9):e10105. doi: 10.15252/msb.202010105 (PMC8444284; doi:10.15252/msb.202010105)
Supplement: Supplementary file 6 — Source Data for Figure 5 [file MSB-17-e10105-s004.zip › Figure5A_sourcedata/GSEA_3017/hallmarks_stateF.GseaPreranked.1621934628066/HALLMARK_G2M_CHECKPOINT.html]

Details for gene set HALLMARK\_G2M\_CHECKPOINT[GSEA]

|  || Dataset | state33017 |
| Phenotype | NoPhenotypeAvailable |
| Upregulated in class | na\_neg |
| GeneSet | HALLMARK\_G2M\_CHECKPOINT |
| Enrichment Score (ES) | -0.758601 |
| Normalized Enrichment Score (NES) | -5.0077934 |
| Nominal p-value | 0.0 |
| FDR q-value | 0.0 |
| FWER p-Value | 0.0 |
Table: GSEA Results Summary

  

Fig 1: Enrichment plot: HALLMARK\_G2M\_CHECKPOINT      
 Profile of the Running ES Score & Positions of GeneSet Members on the Rank Ordered List

  

| PROBE | GENE SYMBOL | GENE\_TITLE | RANK IN GENE LIST | RANK METRIC SCORE | RUNNING ES | CORE ENRICHMENT || 1 | RPS6KA5 |  |  | 438 | 0.456 | -0.1995 | No |
| 2 | SLC12A2 |  |  | 782 | 0.351 | -0.3558 | No |
| 3 | ATRX |  |  | 793 | 0.348 | -0.3562 | No |
| 4 | HIF1A |  |  | 972 | 0.314 | -0.4357 | No |
| 5 | ARID4A |  |  | 984 | 0.312 | -0.4371 | No |
| 6 | FOXN3 |  |  | 1126 | 0.288 | -0.4996 | No |
| 7 | DMD |  |  | 1153 | 0.285 | -0.5083 | No |
| 8 | MEIS1 |  |  | 1407 | 0.256 | -0.6236 | No |
| 9 | NOTCH2 |  |  | 1420 | 0.255 | -0.6261 | No |
| 10 | CUL3 |  |  | 1483 | -0.253 | -0.6520 | No |
| 11 | RPA2 |  |  | 1490 | -0.256 | -0.6517 | No |
| 12 | NOLC1 |  |  | 1542 | -0.270 | -0.6723 | No |
| 13 | E2F1 |  |  | 1580 | -0.281 | -0.6862 | No |
| 14 | CDKN2C |  |  | 1593 | -0.285 | -0.6883 | No |
| 15 | POLA2 |  |  | 1622 | -0.291 | -0.6979 | No |
| 16 | GSPT1 |  |  | 1653 | -0.303 | -0.7082 | No |
| 17 | MAP3K20 |  |  | 1694 | -0.317 | -0.7231 | No |
| 18 | MCM3 |  |  | 1738 | -0.335 | -0.7391 | No |
| 19 | PLK4 |  |  | 1740 | -0.336 | -0.7355 | No |
| 20 | STIL |  |  | 1744 | -0.337 | -0.7328 | No |
| 21 | TOP1 |  |  | 1749 | -0.340 | -0.7305 | No |
| 22 | SMARCC1 |  |  | 1810 | -0.369 | -0.7541 | Yes |
| 23 | NEK2 |  |  | 1815 | -0.371 | -0.7514 | Yes |
| 24 | NUP50 |  |  | 1823 | -0.376 | -0.7501 | Yes |
| 25 | SAP30 |  |  | 1826 | -0.378 | -0.7464 | Yes |
| 26 | CDC45 |  |  | 1836 | -0.382 | -0.7460 | Yes |
| 27 | BARD1 |  |  | 1839 | -0.384 | -0.7422 | Yes |
| 28 | SRSF10 |  |  | 1851 | -0.390 | -0.7426 | Yes |
| 29 | SQLE |  |  | 1863 | -0.396 | -0.7430 | Yes |
| 30 | SYNCRIP |  |  | 1877 | -0.403 | -0.7441 | Yes |
| 31 | CBX1 |  |  | 1878 | -0.405 | -0.7392 | Yes |
| 32 | SRSF1 |  |  | 1879 | -0.405 | -0.7342 | Yes |
| 33 | POLQ |  |  | 1891 | -0.417 | -0.7343 | Yes |
| 34 | NSD2 |  |  | 1900 | -0.425 | -0.7329 | Yes |
| 35 | DKC1 |  |  | 1904 | -0.428 | -0.7291 | Yes |
| 36 | E2F3 |  |  | 1907 | -0.429 | -0.7248 | Yes |
| 37 | SFPQ |  |  | 1908 | -0.429 | -0.7195 | Yes |
| 38 | HMGA1 |  |  | 1915 | -0.433 | -0.7170 | Yes |
| 39 | KMT5A |  |  | 1919 | -0.437 | -0.7131 | Yes |
| 40 | GINS2 |  |  | 1932 | -0.445 | -0.7133 | Yes |
| 41 | STAG1 |  |  | 1943 | -0.450 | -0.7125 | Yes |
| 42 | CCNF |  |  | 1947 | -0.451 | -0.7084 | Yes |
| 43 | DDX39A |  |  | 1957 | -0.458 | -0.7070 | Yes |
| 44 | NASP |  |  | 1988 | -0.481 | -0.7152 | Yes |
| 45 | CDC6 |  |  | 1991 | -0.483 | -0.7102 | Yes |
| 46 | TRA2B |  |  | 1998 | -0.490 | -0.7071 | Yes |
| 47 | XPO1 |  |  | 2007 | -0.500 | -0.7047 | Yes |
| 48 | CTCF |  |  | 2010 | -0.502 | -0.6995 | Yes |
| 49 | CHEK1 |  |  | 2013 | -0.504 | -0.6943 | Yes |
| 50 | SMC1A |  |  | 2021 | -0.513 | -0.6913 | Yes |
| 51 | JPT1 |  |  | 2036 | -0.525 | -0.6915 | Yes |
| 52 | NCL |  |  | 2045 | -0.532 | -0.6887 | Yes |
| 53 | TROAP |  |  | 2046 | -0.533 | -0.6822 | Yes |
| 54 | TTK |  |  | 2047 | -0.533 | -0.6757 | Yes |
| 55 | ILF3 |  |  | 2052 | -0.537 | -0.6710 | Yes |
| 56 | KIF22 |  |  | 2053 | -0.541 | -0.6644 | Yes |
| 57 | INCENP |  |  | 2061 | -0.556 | -0.6609 | Yes |
| 58 | STMN1 |  |  | 2063 | -0.567 | -0.6545 | Yes |
| 59 | RACGAP1 |  |  | 2072 | -0.579 | -0.6512 | Yes |
| 60 | CENPA |  |  | 2073 | -0.586 | -0.6440 | Yes |
| 61 | BUB3 |  |  | 2078 | -0.596 | -0.6386 | Yes |
| 62 | AURKB |  |  | 2079 | -0.598 | -0.6313 | Yes |
| 63 | PLK1 |  |  | 2087 | -0.624 | -0.6270 | Yes |
| 64 | HNRNPU |  |  | 2090 | -0.628 | -0.6202 | Yes |
| 65 | BRCA2 |  |  | 2100 | -0.650 | -0.6165 | Yes |
| 66 | SNRPD1 |  |  | 2105 | -0.653 | -0.6104 | Yes |
| 67 | KPNB1 |  |  | 2108 | -0.660 | -0.6033 | Yes |
| 68 | AURKA |  |  | 2110 | -0.673 | -0.5956 | Yes |
| 69 | CKS2 |  |  | 2115 | -0.694 | -0.5890 | Yes |
| 70 | CDC20 |  |  | 2117 | -0.695 | -0.5810 | Yes |
| 71 | RAD21 |  |  | 2118 | -0.697 | -0.5724 | Yes |
| 72 | SRSF2 |  |  | 2122 | -0.706 | -0.5652 | Yes |
| 73 | HNRNPD |  |  | 2126 | -0.713 | -0.5579 | Yes |
| 74 | ODC1 |  |  | 2128 | -0.718 | -0.5496 | Yes |
| 75 | KIF2C |  |  | 2136 | -0.756 | -0.5437 | Yes |
| 76 | KIF5B |  |  | 2137 | -0.756 | -0.5345 | Yes |
| 77 | NDC80 |  |  | 2142 | -0.775 | -0.5269 | Yes |
| 78 | FBXO5 |  |  | 2144 | -0.779 | -0.5178 | Yes |
| 79 | KIF23 |  |  | 2145 | -0.782 | -0.5083 | Yes |
| 80 | HMGB3 |  |  | 2164 | -0.852 | -0.5063 | Yes |
| 81 | KIF20B |  |  | 2165 | -0.852 | -0.4959 | Yes |
| 82 | DTYMK |  |  | 2168 | -0.858 | -0.4864 | Yes |
| 83 | BUB1 |  |  | 2170 | -0.860 | -0.4764 | Yes |
| 84 | LBR |  |  | 2173 | -0.870 | -0.4667 | Yes |
| 85 | KPNA2 |  |  | 2178 | -0.894 | -0.4577 | Yes |
| 86 | HMMR |  |  | 2179 | -0.895 | -0.4467 | Yes |
| 87 | CCNA2 |  |  | 2181 | -0.902 | -0.4362 | Yes |
| 88 | CHAF1A |  |  | 2182 | -0.903 | -0.4252 | Yes |
| 89 | KIF15 |  |  | 2185 | -0.915 | -0.4150 | Yes |
| 90 | KIF4A |  |  | 2186 | -0.921 | -0.4037 | Yes |
| 91 | ORC6 |  |  | 2188 | -0.937 | -0.3928 | Yes |
| 92 | LMNB1 |  |  | 2194 | -0.950 | -0.3835 | Yes |
| 93 | CDKN3 |  |  | 2199 | -0.979 | -0.3734 | Yes |
| 94 | CCNB2 |  |  | 2200 | -0.983 | -0.3614 | Yes |
| 95 | CKS1B |  |  | 2201 | -0.987 | -0.3494 | Yes |
| 96 | SMC2 |  |  | 2205 | -1.006 | -0.3385 | Yes |
| 97 | KIF11 |  |  | 2206 | -1.008 | -0.3262 | Yes |
| 98 | EZH2 |  |  | 2207 | -1.017 | -0.3138 | Yes |
| 99 | CENPE |  |  | 2211 | -1.036 | -0.3026 | Yes |
| 100 | KNL1 |  |  | 2213 | -1.054 | -0.2902 | Yes |
| 101 | MYBL2 |  |  | 2215 | -1.058 | -0.2777 | Yes |
| 102 | UBE2C |  |  | 2218 | -1.081 | -0.2655 | Yes |
| 103 | CDK1 |  |  | 2225 | -1.153 | -0.2542 | Yes |
| 104 | PTTG1 |  |  | 2226 | -1.198 | -0.2396 | Yes |
| 105 | MAD2L1 |  |  | 2228 | -1.204 | -0.2254 | Yes |
| 106 | UBE2S |  |  | 2229 | -1.205 | -0.2106 | Yes |
| 107 | DBF4 |  |  | 2232 | -1.247 | -0.1964 | Yes |
| 108 | HMGN2 |  |  | 2233 | -1.267 | -0.1809 | Yes |
| 109 | SMC4 |  |  | 2236 | -1.281 | -0.1662 | Yes |
| 110 | PBK |  |  | 2237 | -1.325 | -0.1500 | Yes |
| 111 | TMPO |  |  | 2239 | -1.382 | -0.1336 | Yes |
| 112 | PRC1 |  |  | 2240 | -1.397 | -0.1166 | Yes |
| 113 | BIRC5 |  |  | 2242 | -1.469 | -0.0991 | Yes |
| 114 | TOP2A |  |  | 2246 | -1.562 | -0.0815 | Yes |
| 115 | NUSAP1 |  |  | 2247 | -1.600 | -0.0620 | Yes |
| 116 | TPX2 |  |  | 2248 | -1.609 | -0.0423 | Yes |
| 117 | CENPF |  |  | 2249 | -1.691 | -0.0217 | Yes |
| 118 | MKI67 |  |  | 2252 | -1.892 | 0.0005 | Yes |
Table: GSEA details [plain text format]

  

Fig 2: HALLMARK\_G2M\_CHECKPOINT: Random ES distribution      
 Gene set null distribution of ES for **HALLMARK\_G2M\_CHECKPOINT**
